# Significantly Enhanced Electromechanical Performance of PDMS Crosslinked PVDF Hybrids

**DOI:** 10.3390/polym10070714

**Published:** 2018-06-29

**Authors:** Dan He, Yunchuan Xie, Xiao Wang, Zhicheng Zhang

**Affiliations:** Department of Materials Chemistry, School of Science, Xi’an Key Laboratory of Sustainable Energy Materials Chemistry, Xi’an Jiaotong University, No. 28 Xianning West Road, Xi’an 710049, China; h15872670541@163.com (D.H.); yogurt_wx@stu.xjtu.edu.cn (X.W.); zhichengzhang@mail.xjtu.edu.cn (Z.Z.)

**Keywords:** PVDF-based polymers, electromechanical performance, silicone crosslinked hybrid, electroactive polymers, ferroelectric polymers

## Abstract

Poly(vinylidene fluoride)-based ferroelectric polymers have large and tunable dielectric permittivity (*ε*_r_), but rather high Young’s modulus (*Y*), which limits its electromechanical response when used as actuators. In this work, a silicone oligomer involving amino groups is employed to crosslink a non-crystallized poly(vinylidene fluoride-chlorotrifluoroethylene) matrix bearing double bonds (P(VDF-CTFE-DB)) via addition reaction. Thanks to the flexible silicone molecules, the modulus of the hybrids is reduced over 30% when compared with the pristine matrix. Most interestingly, the *ε*_r_ of the hybrids is improved to nearly 100% higher than that of the matrix when the silicone content reaches 30 wt %. This may be due to the dilution effect of silicone molecules, which favors macromolecular chain rearrangement and dipole orientation of the hybrids under an applied electric field. As a result, electric-field activated displacements of the above hybrid increases to 0.73 mm from 0.48 mm of the matrix under 60 MV/m. The maximum electric field-induced thickness strain increases from 1% of the matrix to nearly 3% of the crosslinked hybrid. This work may provide a facile strategy to fabricate PVDF-based hybrids with enhanced electromechanical performance under low activating voltage.

## 1. Introduction

As an important branch of electro-active polymers (EAPs), dielectric elastomer (DE) has the merits of high electromechanical efficiency, large deformation, and fast response times, which has potential application in the fields of artificially intelligent and wearable devices, such as actuators, nano-generators, and biosensors [1,2,3,4,5,6,7,8]. DEs’ shape and volume may change when exposed to an external electric field, accompanied by the conversion between electrical and mechanical energy. For the film-shaped samples, the electric field-induced thickness strain (*S*_z_) could be determined by polarization (*P*) under the electric field and Young’s modulus (*Y*) of the elastomer, e.g., $S_{Z}=-\frac{P}{Y}=-\frac{\varepsilon_{0}\varepsilon_{r}E^{2}}{Y}$, in which *ε*_0_ and *ε*_r_ are the vacuum permittivity (8.85 × 10^−12^ F/m) and dielectric permittivity, respectively, and *E* is the electric field applied on the film sample [9,10,11]. As a result, *ε*_r_*/Y* is defined as an electromechanical efficiency, which characterizes the energy conversation efficiency. Apparently, the higher the *ε*_r_*/Y*, the larger the *S*_z_ may be realized under the given electric field, which means the DEs with high *ε*_r_ and low *Y* values are favorable to obtain large electromechanical strain. Meanwhile, a low activation electric field (*E*_a_) is favorable for elastomers, especially for the wearable devices and medical instruments applications, where the high voltage is prohibited due to intimate contact with the human body.

Fluoropolymer (FP) plays an increasingly important role in the building, automobile, and other industries because of its excellent performance of self-flame-retardancy, self-cleaning, low friction coefficient, high corrosion resistance, and high temperature resistance. Poly(vinylidene fluoride) (PVDF)-based fluoropolymers have been well studied as electrostrictive EAPs for their ferroelectric properties and multiple crystalline morphologies, which results in a tunable *ε*_r_ ranging from 10 to 100, along with rather high breakdown strength (*E*_b_) (100~300 MV/m). Meanwhile, their excellent comprehensive properties, including electrical activity, chemical and weather resistance, as well as biocompatibility, assure their applications for actuators dealing with high power (mechanical arm or artificial muscle) and sensors working under severe circumstances (high pressure or high irradiation) [12,13,14,15]. However, PVDF-based FPs represented by P(VDF-TrFE) (poly(vinylidene fluoride-trifluoroethylene)) and P(VDF-TrFE-CTFE)s (CTFE refers to chlorotrifluoroethylene) usually possess a high *Y* of 0.3~0.4 GPa for their semi-crystalline structure, thus delivering a relatively low electromechanical efficiency (*ε*_r_*/Y*). The reported study is mostly focused on improving *ε*_r_ to achieve high electromechanical performance [16,17,18,19,20]. Typically, conductive or high-*ε*_r_ fillers, such as graphite, carbon black, or ferroelectric ceramics (PMN, PLZT, BT, etc.) are introduced into well-known elastomers, such as poly(acrylates), silicone elastomers, and polyurethane (PU) [21,22,23,24,25,26,27,28,29]. However, the addition of high-*ε*_r_ particles inevitably causes the increase of *Y* and the reduced *E*_b_, thus, the undesirable reduction of electromechanical efficiency. Therefore, how to adjust the *ε*_r_*/Y* value of FPs reasonably is crucial to realize high electromechanical performance.

In addition to increasing the permittivity of the elastomers, the decreasing *Y* of high-*ε*_r_ FPs is the other option to improve *ε*_r_*/Y*. In the present work, an amorphous fluoropolymer matrix (P(VDF-CTFE):poly(vinylidene fluoride-chlorotrifluoroethylene)) with relatively low modulus is chosen as the pristine matrix, where the desired -CH=CF- unsaturation is inserted by a dehydrochlorination process catalyzed with triethylamine (TEA) as active sites. A silicone oligomer capped with NH_2_ groups (NH_2_-PDMS-NH_2_) is introduced by the addition reaction between amine and -CH=CF- units, which results in not only the anchored silicone oligomer, but also the cured matrix. As desired, the modulus of the hybrid polymer is significantly reduced thanks to the addition of flexible silicone cross-linkers. Most interestingly, the dielectric permittivity of the hybrid is enhanced nearly 100% compared to the pristine matrix. Both the reduced *Y* and the improved *ε*_r_ are responsible for the tripled strain of the optimized hybrid DEs. This work may provide a facile strategy to fabricate PVDF-based dielectrics with large electromechanical strain and low actuating voltage.

## 2. Materials and Methods

### 2.1. Materials

P(VDF-CTFE) (VDF/CTFE = 80/20 in molar ratio, *M*_n_ = 120,000 g/mol, polydispersity index (PDI) = 2.20) was provided by Zhonghao Chenguang Research Institute of Chemical Industry (Chengdu, China). Low molecular weight silane oligomer with amino groups (NH_2_-PDMS-NH_2_) was purchased from Silok Chemicals Co. Ltd. (Guangzhou, China) and used as received. Triethylamine (TEA), ethyl acetate (EAc), *N*-methyl pyrrolidone (NMP) and anhydrous ethanol were provided by Tianjin Chemical Reagent Co. Ltd. (AR grade, Tianjin, China).

### 2.2. Synthesis of P(VDF-CTFE-DB) Matrix

The polymer matrix, poly(vinylidene fluoride-chlorotrifluoroethylene) bearing double bonds (P(VDF-CTFE-DB)) (VDF/CTFE/DB = 68/8/12 in molar ratio), was synthesized from P(VDF-CTFE) (VDF/CTFE = 80/20 in molar ratio) by a controlled dehydrochlorination process as follows [30]: Into a 150 mL double-necked flask equipped with condenser and magnetic stirrer, 2.0 g P(VDF-CTFE) and 60 mL NMP were charged in sequence. After the polymer was dissolved completely, 0.54 g triethylamine was added and the elimination reaction was conducted at 50 °C under vigorous stirring for 24 h. The solution was slowly poured into 500 mL DI water and the resultant polymer was precipitated and collected by washing the precipitant with DI water three times and with methanol twice before drying at 45 °C for 48 h.

### 2.3. Preparation of Silicone Cross-Linked P(VDF-CTFE-DB) Hybrids

**Figure 1:**
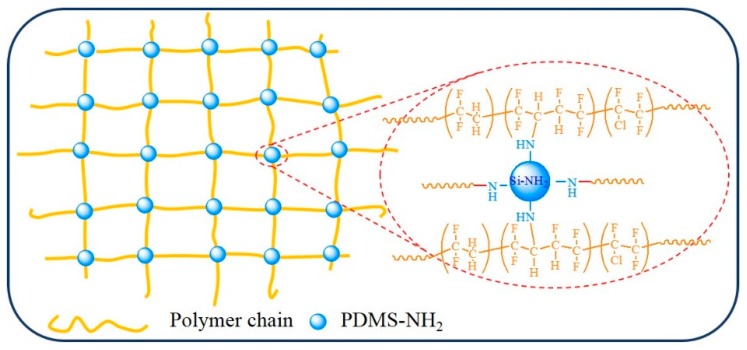
Schematic illustration for the chemical structure of the hybrid.

Silicone oligomer crosslinked P(VDF-CTFE-DB) hybrid films with a thickness of ~50 μm (+/− 5 μm) were prepared via a typical solution cast process [31,32] on glass plates and the structure of the hybrids was illustrated in Figure 1. P(VDF-CTFE-DB) matrix and silicone oligomers were dispersed into EAc by vigorously stirring for 24 h to form a transparent solution with concentration of 5 wt %. The hybrid films were fabricated by casting the filtered solution bearing silicone of varied weight contents and polymer matrix onto glass slides, followed by removing all the volatiles at 80 °C for eight hours. The increased weight contents of silicone, 0.0, 10.0, 20.0, 30.0, 40.0, and 50.0 wt %, were designed in the resultant hybrids.

For expression convenience, the materials and hybrids used in the present work are indicated by abbreviations as listed in Table 1.

### 2.4. Characterizations

Fourier-transform infrared (FTIR) spectra were recorded on an IR spectrophotometer (Bruker-Tensor 27, Karlsruhe, Germany) with a resolution of 2 cm^−1^ and 16 scans. X-ray diffraction (XRD) measurement was conducted on a Rigaku D/max 2400 diffractometer (Rigaku Industrial Corp., Osaka, Japan) with the X-ray wavelength of 1.542 Å (Cu Kα radiation, 40 kV and 100 mA), the 2θ diffraction angle was from 20° to 80°, at a rate of 15°/min, and a step of 0.02°. Scanning electron microscopy (FE-SEM, JEOL Ltd., Tokyo, Japan) observation was performed on a JSM-2400, and the samples were fractured with liquid nitrogen and coated with a thin layer of gold prior to the observation.

Differential scanning calorimetry (DSC) measurements were carried out on a Netzsh PC-200 DSC (Bavaria, Germany), and the calorimeter had been calibrated with indium before use. All the measurements were carried out under nitrogen atmosphere from room temperature to 180 °C at a heating rate of 10 °C/min. Thermogravimetric analysis (TGA) was tested on a STA 449F3 (Netzsch, Bavaria, Germany) at a heating rate of 10 °C/min under N_2_ flow. The elastic modulus was determined by a commercial DMA-Q800 analyzer (TA Instruments, New Castle, DE, USA). Specimens were fabricated into sheets with a size of 35 mm × 10 mm × 0.1 mm. Proton nuclear magnetic resonance (^1^H-NMR) spectra were carried out on a Bruker (Advance III, Karlsruhe, Germany) 400 MHz spectrometer with acetone-d6 as the solvent and tetramethylsilane as an internal standard.

The dielectric properties and AC electrical conductivity of all hybrid films were obtained via a HP4284A LCR meter (Agilent Technologies, Santa Clara, CA, USA) at frequencies ranging from 100 Hz to 1 MHz with 1 V voltage bias at room temperature. Gold electrodes with a diameter of 3 mm and a thickness around 30 nm were sputtered on both sides of the hybrid films by JEOL JFC-1600 auto fine coater (Tokyo, Japan) for all electrical properties’ characterizations. The electric field-induced electromechanical response, including the thickness strain and tip displacement of the films, was measured by using a laser distance-detecting instrument (LK-G150, Keyence, Osaka, Japan) and high voltage amplifier.

## 3. Results and Discussion

### 3.1. Chemical Structure of P(VDF-CTFE) and P(VDF-CTFE-DB)

**Figure 2:**
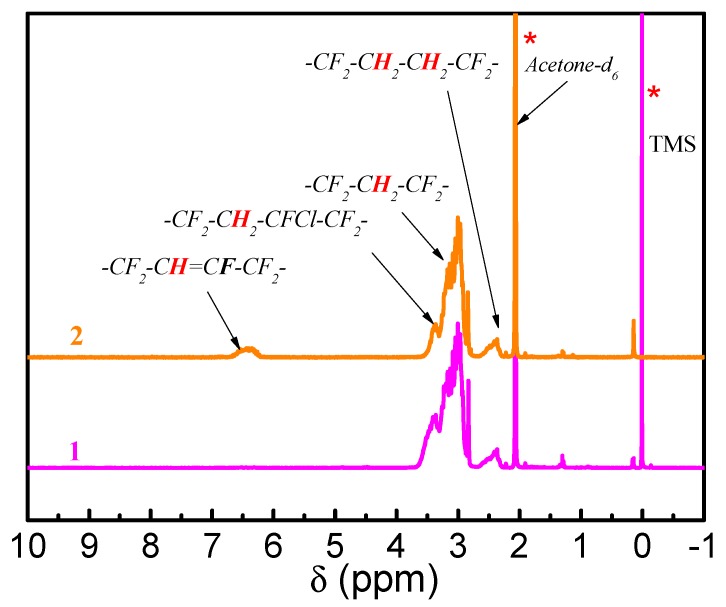
^1^H-NMR spectra of P(VDF-CTFE) (Line 1) and P(VDF-CTFE-DB) (Line 2).

The chemical composition of P(VDF-CTFE-DB) is confirmed with ^1^H-NMR as shown in Figure 2. Resonance signals at 2.2–2.7 ppm and 2.7–3.2 ppm are assigned to head-head (-CF_2_-C*H*_2_-C*H*_2_-CF_2_-) and head-tail (-CF_2_-C*H*_2_-CF_2_-CH_2_-) connections of VDF units. The signal at 3.2–3.6 ppm is identified as protons on VDF units adjacent to CTFE units (-CF_2_-C*H*_2_-CFCl-CF_2_-). Compared to P(VDF-CTFE), new multiple signals at 6.2–6.7 ppm are assigned to protons on double bonds (-CF_2_-*CF*=*CH*-CF_2_-) after the removal of HCl from (-CF_2_-*CFCl-CH*_2_-CF_2_-) [33].

**Scheme 1:**
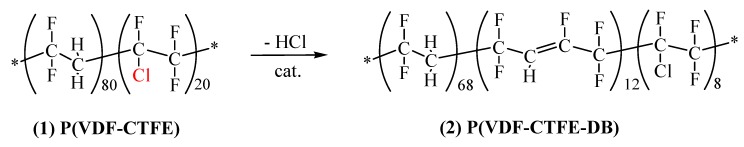
Synthesis route of P(VDF-CTFE-DB) from P(VDF-CTFE).

**Figure 3:**
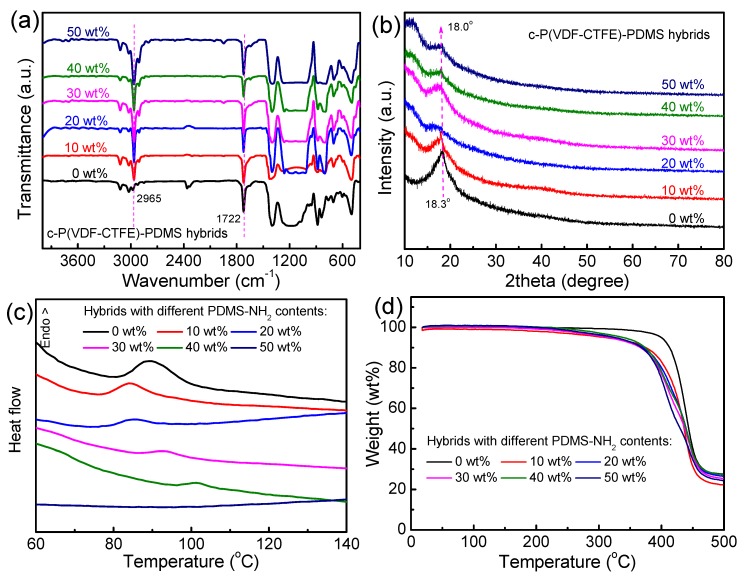
(**a**) FT-IR spectra, (**b**) WAXD patterns, (**c**) DSC curves, and (**d**) TGA result of the hybrids with various PDMS-NH_2_ contents.

Based on the integral calculation, the chemical composition is estimated to be 68 mol % VDF, 8 mol % CTFE, and 12 mol % DB (-CF_2_-CF=C*H*-CF_2_-), as illustrated in Scheme 1. Moreover, the successful introduction of -CF=CH- bonds could be further confirmed by characteristic stretching vibration absorption of C=C bonds at 1722 cm^−1^ and out-plane bending vibration signal of C-H bonds in -CF=CH- at 704 cm^−1^ through the FT-IR results of P(VDF-CTFE-DB), as presented in Figure 3a.

### 3.2. Structure and Morphology of the Hybrids

The chemical structure and thermal properties of the composites are characterized with FT-IR, XRD, DSC, and TGA, respectively. The FTIR spectra of pure matrix and the hybrids with silicone content ranging from 10 wt % to 50 wt % are shown in Figure 3a. Compared with the pristine P(VDF-CTFE-DB) matrix, no particular new absorption bands occur in the spectra of the hybrids, which means that the introduction of silicone molecules would not alter the chain configuration or crystal form of the polymer matrix. However, the obviously-increased intensity of absorption at ~2965 cm^−1^ against increasing PDMS-NH_2_ content is assigned to the stretching vibration of C-H bonds on –CH_3_, which confirmed the successful introduction of silicone molecules.

XRD patterns of the matrix and the hybrids are plotted in Figure 3b. All the patterns show no sharp crystalline peaks in a wide measuring 2theta range suggesting both the matrix and the hybrids are in a totally amorphous phase. The peak at about 18 °C with wide and dispersing shape might be the diffraction of the locally-ordered structure within the amorphous polymer, which may be formed from the strong coupling effect between high polar VDF dipoles. Diffraction intensity of the peak decreases gradually with the increasing amount of silicone suggesting the weakened dipoles’ coupling effect. In addition, a minor shift of the 2theta peak from 18.3° of pure matrix to 18.0° of c-P(VDF-CTFE)-PDMS containing 50 wt % PDMS is observed, which implies the enlarged chain distance of PVDF matrix by the dilution of PDMS. DSC results presented in Figure 3c show no visible melting transition on heating circles and the broad endothermic peak between 80 to 110 °C may ascribed to the relaxation process of the locally-ordered structure, as mentioned above. Apparently, the crosslinked structure introduced by PDMS addition could impede the ordered arrangements of the VDF units, which, in turn, depress the relaxation process as discussed. The DSC results agree well with that of the XRD measurements. TGA results presented in Figure 3d suggest that the addition of PDMS crosslinking leads to the reduced initial decomposition temperature (Ts) of the hybrid, which is about 50 °C lower than the pristine matrix. This may be attributed to the lower thermal stability of PDMS than PVDF. All the results suggest that the resultant materials contains two different components. 

**Figure 4:**
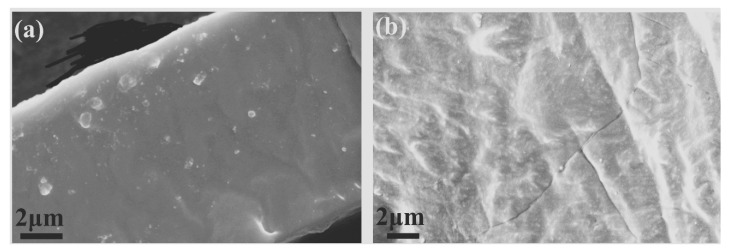
SEM images of (**a**) the pristine matrix and (**b**) the hybrid with 30 wt % PDMS-NH_2_.

The cross-sections of matrix and the hybrids are observed through SEM. As presented in Figure 4, both samples show rough surface morphologies with ravine-like structures, which suggest their typical ductile fractures. However, compared with the even texture of the matrix, obvious uneven surface morphology, e.g., the “sea-island” like structure is detected in the hybrid. This may be attributed to the low compatibility of the fluoropolymer main chain and PDMS segment, which may form a visible phase separation structure in the hybrid with high PDMS loading content.

### 3.3. Dielectric Properties of the Hybrids

**Figure 5:**
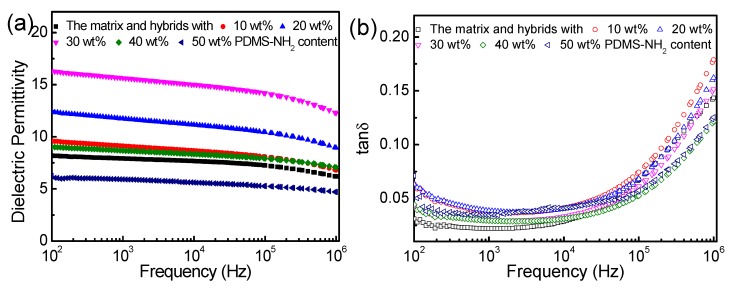
Frequency dependence of (**a**) the dielectric permittivity and (**b**) the loss tangent of the neat matrix and the hybrids with the indicated PDMS-NH_2_ contents under room temperature.

The frequency dependence of dielectric permittivity of the hybrids measured on a HP4284A LCR instrument at room temperature is shown in Figure 5a. The dielectric permittivity of all the hybrids decreases gradually as a function of the frequency ranging from 100 Hz to 1 MHz, owing to the reduced dipole response, which is similar to the matrix. Due to the dilution effect of PDMS, *ε*_r_ of the hybrids is enhanced with the increasing content of PDMS until 30 wt % owing to the reduced coupling forces among dipoles. For example, the permittivity of the hybrid containing 30 wt % PDMS (~16) is almost doubled with respect to eight of the pristine matrices at 100 Hz. However, when PDMS content is over 30 wt %, *ε*_r_ of the hybrids starts to decrease, which may contribute to the dilution effect of low-*ε*_r_ PDMS. The frequency dependence of the dielectric loss (or tanδ) of the hybrids is plotted in Figure 5b. The dielectric loss agrees well with the permittivity results, namely, the addition of PDMS below 30 wt % leads to the increased tanδ at high frequency. When the PDMS content is over 30 wt %, the dielectric loss is reduced as well.

### 3.4. Mechanical Properties of the Hybrids

**Figure 6:**
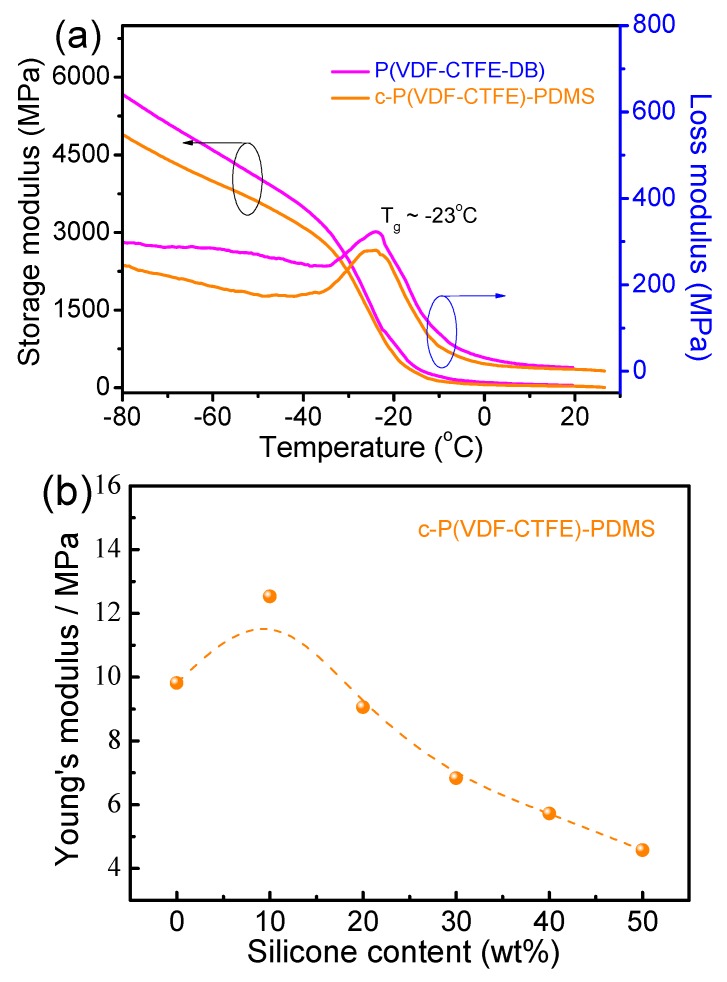
(**a**) DMA curves of neat matrix and hybrid containing 30 wt % PDMS-NH_2_, and (**b**) the modulus of the hybrids with various silicone contents at room temperature.

To evaluate the effects of PDMS on the mechanical modulus of the matrix, DMA measurements are performed and the results are shown in Figure 6. As temperature increases, the modulus of P(VDF-CTFE-DB) decreases slowly and a quick drop is observed at about −25 °C corresponding to the glass transition temperature (Tg). The addition of PDMS with certain content leads to continuously reduced both storage and loss modulus in the resultant hybrids, especially at temperatures below Tg. This means the addition of PDMS with low Tg is effectively to soften the PVDF matrix at low temperature. At room temperature, the increased Young’s modulus in the hybrid containing 10 wt % PDMS, as shown in Figure 6b, might be ascribed to the crosslinking of the P(VDF-CTFE) matrix caused by the additional reaction of amine groups onto PDMS. Further increasing the content of PDMS leads to the continuously-reduced modulus thanks to the dilution effect of low-Tg PDMS. 

The dielectric constant, Young’s modulus, and *ε*_r_*/Y* of hybrids bearing varied PDMS are listed in Table 2, from which we can see that electromechanical efficiency (*ε*_r_*/Y*) of the hybrids reaches a maximum value of 2.40 at PDMS content of 30 wt %, which is nearly 200% higher than that of the P(VDF-CTFE-DB) matrix. As suggested by the above-mentioned equation, the thickness strain of the hybrid should be improved accordingly.

### 3.5. Electromechanical Performance of the Hybrids

**Figure 7:**
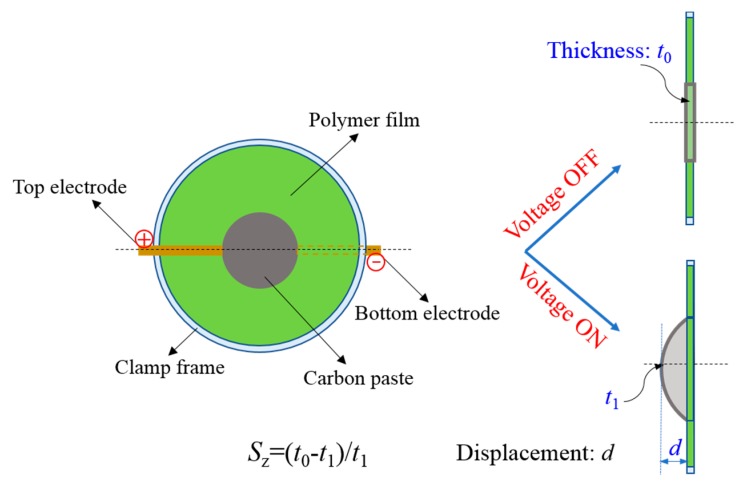
Schematic illustration for measurement of the electromechanical response.

Figure 7 presents the measuring illustration of electromechanical response using circular actuators. After the electric field is applied on the film area coated with carbon paste as the electrode, the electrostatic attraction between the opposite charges of both electrodes may generate an electrostatic stress or pressure on this area, causing the thickness reduction (thickness strain) with the expansion in area. The electric field-induced thickness strain can be measured by a laser detector.

Apparently, in order to obtain materials with large thickness strains, the dielectrics should possess high *ε*_r_, but low *Y* at the same time, or, a large *ε*_r_*/Y* value. In addition, enough high *E*_b_ is also necessary for the dielectrics to work stably under a high electric field.

**Figure 8:**
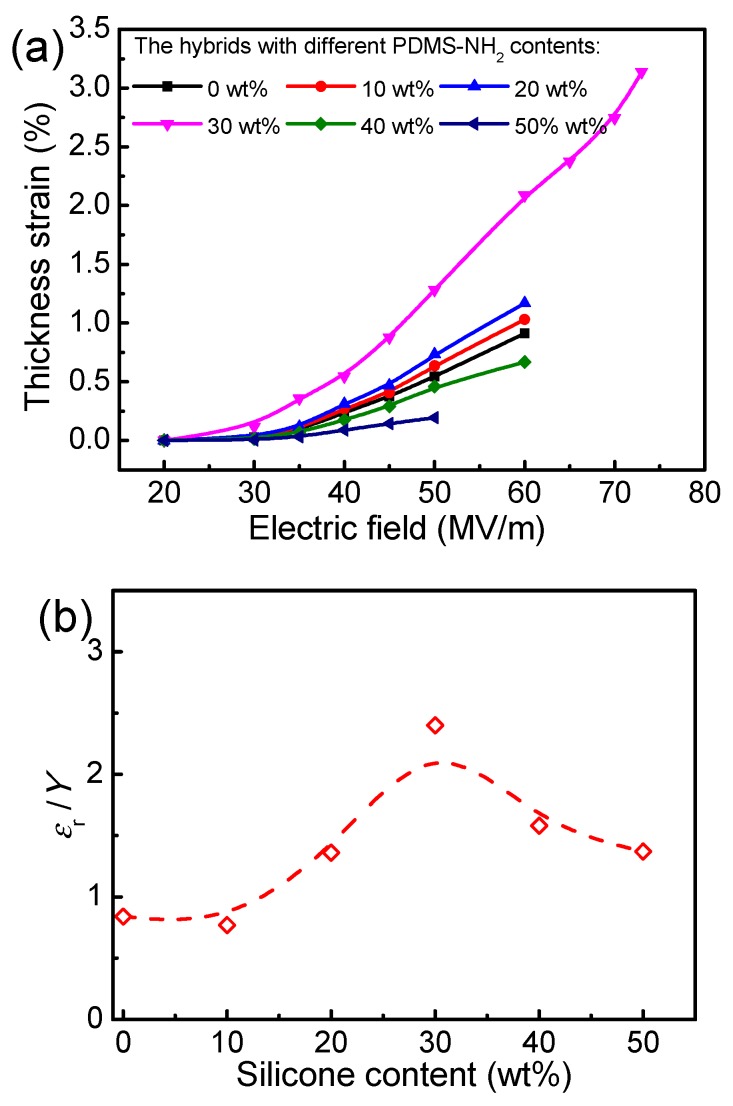
(**a**) Thickness strains of hybrids containing various silicone contents as a function of the electric field under room temperature, and (**b**) the maximum displacements and electromechanical efficiency of the hybrids with various silicone contents.

The measured *S*_z_ for the pristine matrix and the hybrids against the increasing electric field are shown in Figure 8a. Clearly, *S*_z_ of all the samples increases with the elevation of the electric field, which agrees well with equation mentioned in introduction part. Meanwhile, when the silicone content is less than 30 wt %, *S*_z_ under the consistent electric field increases with the increasing silicone contents. For instance, *S*_z_ values of neat matrix and hybrids containing 10 wt %, 20 wt %, and 30 wt % silicone are 0.5%, 0.6%, 0.7%, and 1.3%, respectively, under 50 MV/m. This suggests the addition of silicone molecules could effectively weaken the intermolecular interactions, thus increasing the mobility of molecular dipoles and decrease the modulus of the hybrids (as indicated in Table 2). However, when the silicone content is beyond 30 wt %, *S*_z_ starts to decrease. For example, *S*_z_ values of hybrids containing 40 wt % and 50 wt % silicone decrease to 0.5% and 0.2% under the same electric field of 50 MV/m. This may be ascribed to the dilution effect of PDMS-NH_2_ molecule with low-*ε*_r_ and low *Y* into the matrix, which, in turn, leads to decreased *ε*_r_*/Y* values.

Most interestingly, a maximum *S*_z_ value of over 3.0% is obtained for hybrids containing 30 wt % PDMS under ~75 MV/m, which is nearly two times higher than that of the pristine matrix (0.9%). The significantly enhanced strain can be attributed to the following factors: On one hand, the introduction of flexible silicone oligomers (no more than 30 wt %) can improve thw electromechanical efficiency (*ε*_r_*/Y*) values of the hybrids, as indicated in Figure 8b. On the other hand, high insulating silicone component (~10^14^ Ω·cm) can help improve the electric resistance of the hybrid, thus leading to higher breakdown strength (*E*_b_). It is worth pointing out that all the above tests were performed without any pre-stretching.

**Figure 9:**
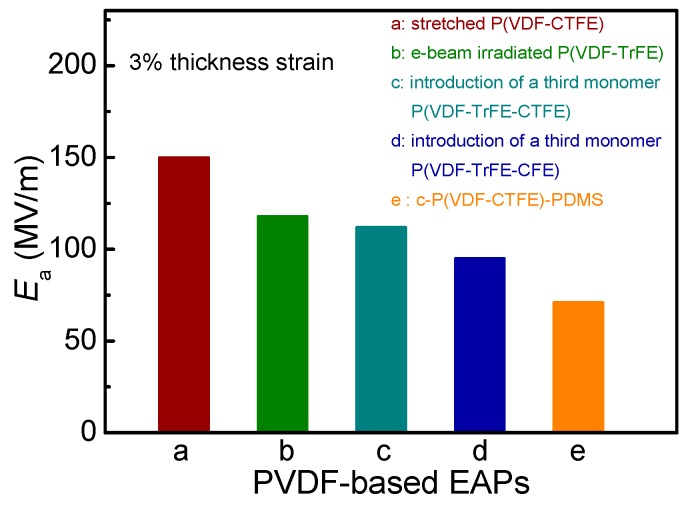
Comparison of *E*_a_ for different PVDF-based EAPs at the 3% thickness strain level.

In order to evaluate the electromechanical conversion ability of c-P(VDF-CTFE)-PDMS hybrids prepared in this work, actuating fields (*E*_a_) need to produce a 3% thickness strain are compared with other reported PVDF-based EAPs, including stretched P(VDF-CTFE) [34], e-beam crosslinked P(VDF-TrFE) [35], copolymer P(VDF-TrFE-CTFE) [36] and P(VDF-TrFE-CFE) [37,38], and c-P(VDF-CTFE)-PDMS. It can be found from Figure 9 that the *E*_a_ values for the five above-mentioned PVDF-based EAPs produce a 3% thickness strain are 150 MV/m, 118 MV/m, 112 MV/m, 95 MV/m, and 71 MV/m, respectively. The result suggests that c-P(VDF-CTFE)-PDMS hybrids possess the advantage of low actuating voltage as well.

## 4. Conclusions

In this study, a novel hybrid fluoropolymer is prepared by using a flexible silicone oligomer as a chemical cross-linker. The optimized hybrid dielectric indicates a simultaneously-improved dielectric constant and reduced modulus, which is 100% higher and 30% less than that of the pristine matrix, respectively. Therefore, the hybrid may produce a significantly enhanced electromechanical strain under the applied electric field, e.g., 3% thickness strain under 70 MV/m, which is three times that of the pristine fluoropolymer. This work may provide a simple strategy to fabricate PVDF-based EAPs with significantly enhanced electromechanical strain.

## Figures and Tables

**Table 1:** Abbreviations for different materials.

Full Name	Abbreviation
Uncross-linked P(VDF-CTFE-DB)	P(VDF-CTFE-DB)
Cross-linked P(VDF-CTFE-DB)	c-P(VDF-CTFE)-PDMS
Silicone oligomer with -NH_2_ groups	PDMS-NH_2_

**Table 2:** Electromechanical efficiency of the hybrids.

Silicone Content (wt %)	0	10	20	30	40	50
*ε*_r_@100 Hz	8.2	9.6	12.4	16.3	9.0	6.3
*Y*/MPa	9.8	12.5	9.1	6.8	5.7	4.6
*ε*_r_ */Y*	0.84	0.77	1.36	2.40	1.58	1.37
